# Application of self-made rubber drainage strip in the operation of giant lipoma

**DOI:** 10.1093/jscr/rjac449

**Published:** 2022-09-30

**Authors:** Qing Chen, Ping Shao

**Affiliations:** Department of General Surgery, Cheng Fei Hospital, Chengdu, Sichuan Province, China; Department of General Surgery, Cheng Fei Hospital, Chengdu, Sichuan Province, China

**Keywords:** lipoma, drainage, rubber strip

## Abstract

Lipoma is a common benign soft-tissue tumor. Giant lipoma [1] is generally defined as more than 10 cm in length or more than 1000 g in weight. We report a 33-year-old patient with a giant lipoma in the left scapular region. We placed a self-made rubber drainage strip during the operation to control exudate, which is effective in improving incision effusion.

## INTRODUCTION

Giant lipomas are relatively rare. Surgical resection is the only curative option. Generally, a huge cavity will be left after surgical resection of a giant lipoma, and it is particularly important to prevent incision infection and fluid accumulation after surgery. Drainage tube can be placed after surgery, but it will affect the postoperative aesthetics and daily activities of the patient. In this case, a self-made rubber drainage strip is placed after the operation of the giant lipoma, which can not only drain the fluid in the surgical area but also make the patient feel comfortable afterwards.

## CASE REPORT

**Figure 1:**
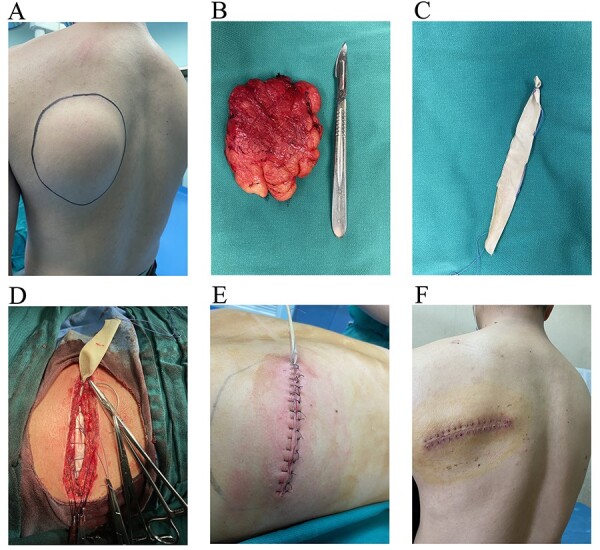
(**A**) A giant mass in the left scapular region. (**B**) A lipoma with a size of about 10.0 cm × 8.7 cm × 1.6 cm. (**C**) A self-made rubber drainage strip. (**D**) Placing a self-made rubber drainage strip before closing the incision. (**E**) Interrupted vertical mattress valgus suture was completed. (**F**) The sutures were removed 12 days postoperatively and the incision healed well.

A 33-year-old man was admitted to our department with complaint of founding a mass in the left scapular region and gradually increased for 2 years, accompanied by discomfort in the supine position. The preoperative imaging examination showed a giant lipoma with a size of about 10.0 cm × 8.7 cm × 1.6 cm. Subsequently, surgical resection was performed. During the operation, we made a transverse fusiform incision on the mass surface and placed a self-made rubber drainage strip before the incision was closed. The rubber drainage strip took from a single-use sterile rubber surgical glove. We pulled out the rubber drainage strip on the second day after the operation. The sutures were removed 12 days postoperatively and the incision healed well (Fig. 1).

## DISCUSSION

Surgical resection of giant lipoma is the most appropriate treatment [2] and not complicated; however, preventing post-surgical complications (the wound fluid or surgical site infection) is of great importance. Due to the relatively large lacuna and unavoidable exudation left after the operation of the giant lipoma, the application of appropriate wound drainage is necessary. We used a self-made rubber drainage strip, which could decrease the wound fluid and surgical site infection after the giant lipoma resection.

## AUTHOR’S CONTRIBUTIONS

Q.C. wrote the manuscript. P.S. drafted the initial manuscript.
